# Omission of axillary surgery in early breast cancer with negative lymph nodes: a systematic review and meta-analysis of randomized clinical trials

**DOI:** 10.1007/s10549-026-07910-y

**Published:** 2026-03-18

**Authors:** Bárbara Bizzo Castelo, Luiz Gustavo Oliveira Brito, Renato Zocchio Torresan, Cássio Cardoso Filho, Giuliano Mendes Duarte

**Affiliations:** State University of Campinas (Unicamp), Campinas, São Paulo, Brazil

**Keywords:** Breast cancer, Axillary surgery, Sentinel lymph node biopsy, Disease-free survival, Randomized clinical trials

## Abstract

**Purpose:**

To evaluate whether the omission of axillary surgery impacts clinical outcomes in patients with early-stage breast cancer and clinically negative lymph nodes.

**Methods:**

We conducted a systematic review and meta-analysis of randomized clinical trials (RCTs) comparing no axillary surgery with standard axillary interventions (sentinel lymph node biopsy [SLNB] or axillary dissection [AD]). This study followed PRISMA guidelines and was registered in PROSPERO (CRD420250653779). Searches were conducted in PubMed, Web of Science, and Embase through June 2025. Outcomes assessed included overall survival (OS), disease-free survival (DFS), and axillary recurrence (AR). Meta-analyses were performed using RevMan 5.4. Risk of bias was assessed using the RoB 2 tool.

**Results:**

Out of 853 records, seven RCTs including 8806 patients met the inclusion criteria. Among them, 2,915 patients underwent no axillary surgery, while 5891 received surgical axillary treatment. Two trials compared no surgery with SLNB, and five compared no surgery with AD. No significant differences were found in OS (OR = 1.02; 95% CI, 0.86–1.20; p = 0.84; I^2^ = 36%) or DFS (OR = 0.80; 95% CI, 0.63–1.00; p = 0.05; I^2^ = 63%). AR was significantly lower in the axillary surgery group (OR = 0.18; 95% CI, 0.10–0.31; p < 0.01; I^2^ = 39%).

**Conclusion:**

The omission of axillary surgery in early-stage breast cancer with clinically negative lymph nodes does not negatively impact overall or disease-free survival. However, it is associated with a higher—though still low—risk of axillary recurrence.

## Introduction

Axillary lymph node status remains one of the most important prognostic factors in breast cancer management [1]. Historically, axillary staging has been achieved through surgical procedures. Since the late nineteenth century, following William S. Halsted’s introduction of the radical mastectomy [2], axillary surgery has played a central role in disease control. However, contemporary axillary management is shifting toward more conservative approaches.

For decades, axillary lymph node dissection (ALND) was the standard method for staging. The introduction of sentinel lymph node biopsy (SLNB) marked a pivotal shift in surgical practice [3, 4]. Compared to ALND, SLNB is associated with significantly lower arm morbidity and better quality of life, becoming the standard of care for early-stage breast cancer with clinically node-negative axillae [5]. Its indications have expanded over time, and ALND can now be safely omitted in selected patients with limited sentinel node involvement [6–10]. SLNB is also considered safe following neoadjuvant chemotherapy, both in initially node-negative patients [11] and in those whose nodal disease has been downstaged [11–13]. These developments reflect a broader trend toward de-escalation of axillary surgery without compromising oncologic outcomes.

The next frontier in this evolution may be the complete omission of axillary surgery. Several randomized clinical trials are currently evaluating the safety of this strategy in selected patients [14–16]. Recently, two trials have reported their primary endpoints, demonstrating that the omission of axillary surgery is non-inferior to SLNB in patients with early-stage breast cancer and clinically and ultrasonographically negative axillae [17, 18]. Nonetheless, whether clinical and imaging assessments can reliably replace surgical staging remains an open question.

This meta-analysis aims to assess whether omitting axillary surgery in patients with early-stage breast cancer and clinically negative lymph nodes impacts clinical outcomes.

## Methods

**Fig. 1:**
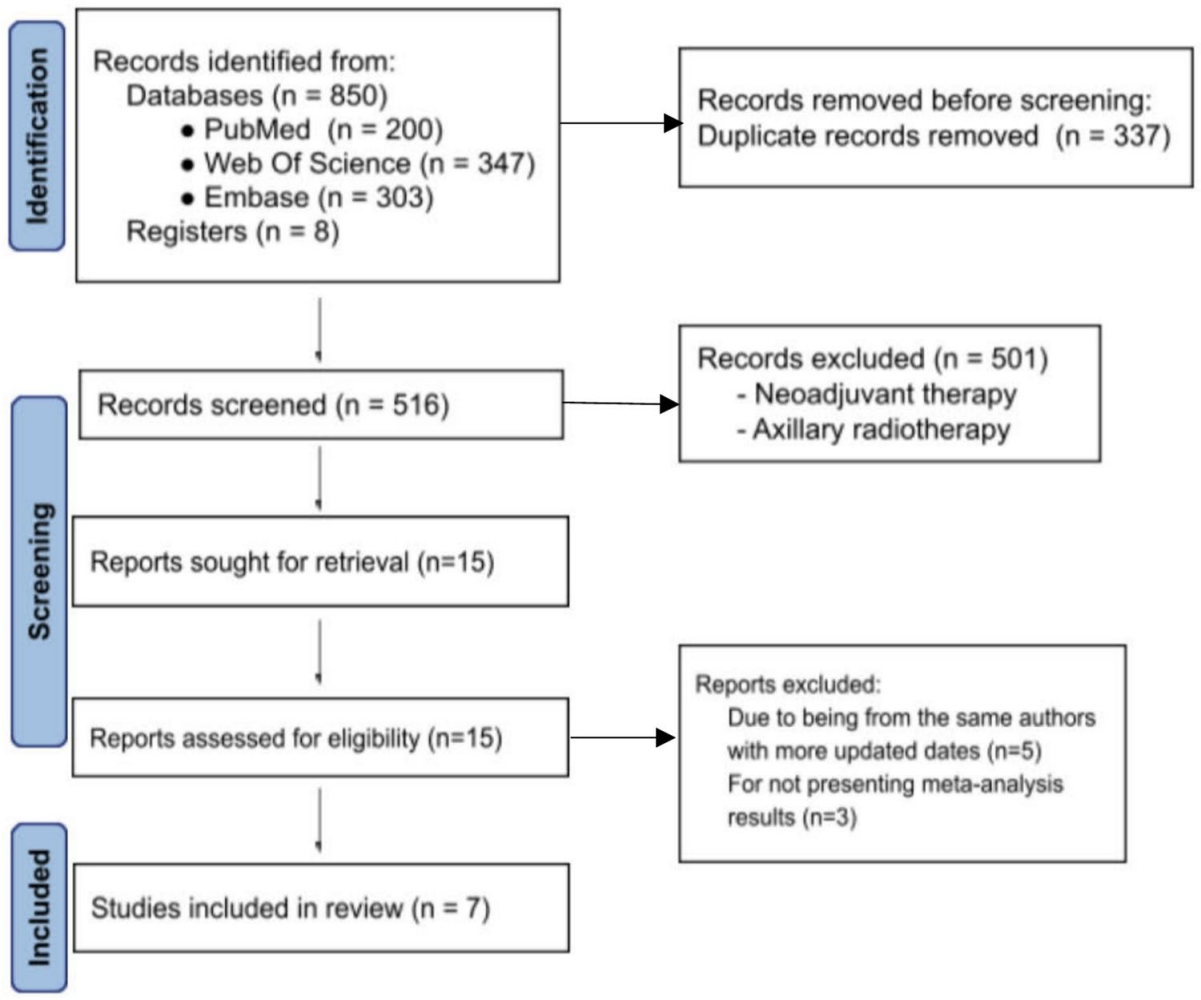
Preferred reporting items for systematic reviews and meta-analyses

This study was conducted in accordance with the Preferred Reporting Items for Systematic Reviews and Meta-Analyses (Fig. 1) guidelines and was registered in PROSPERO (CRD420250653779). We performed a systematic review and meta-analysis of randomized controlled trials (RCTs) comparing omission of axillary surgery versus surgical axillary management (SLNB or ALND) in patients with early-stage breast cancer and clinically and/or radiologically negative axillae.

### Search strategy

Two independent reviewers conducted a systematic search of PubMed, Web of Science, and Embase up to June 2025, using the following MeSH terms: (“breast cancer”) AND (“axillary dissection” OR “axillary surgery” OR “no axillary surgery” OR “sentinel lymph node”) AND (“randomized study” OR “randomized clinical trial”).

### Study selection

Eligible studies were RCTs comparing outcomes between patients who did not undergo axillary surgery and those who underwent SLNB or ALND. The inclusion criteria were: (1) adult women with early-stage breast cancer (T1–T3, N0); (2) negative clinical and/or ultrasound axillary assessment; and (3) reporting of at least one oncologic outcome: overall survival (OS), disease-free survival (DFS) or axillary recurrence (AR). Trials involving neoadjuvant chemotherapy, clinically positive nodes, or axillary radiotherapy were excluded.

### Data extraction and synthesis

Data were independently extracted by two reviewers using a standardized form, with discrepancies resolved by a third reviewer. Extracted variables included study design, sample size, type of intervention, inclusion criteria, follow-up duration, and reported outcomes. The primary outcomes were overall survival, disease-free survival, and axillary recurrence. Study selection was managed using Rayyan software. Meta-analysis was performed with RevMan version 5.4, with dichotomous variables analyzed using odds ratios (ORs) and 95% confidence intervals (CIs). Heterogeneity was assessed using the I^2^ statistic and addressed using a random-effects model.

### Risk of bias assessment

Risk of bias was assessed using the Cochrane Risk of Bias 2 (RoB 2) tool across five domains. The risk of bias for each outcome was summarized qualitatively and illustrated graphically.

## Results

Seven randomized controlled trials (RCTs) were included: Reimer et al., 2024 [17]; Gentilini et al., 2023 [18]; Agresti et al., 2024 [19]; Martelli et al., 2017 [20]; Avril et al., 2012 [21]; Rudenstam et al., 2005 [22]; and Fisher et al., 2002 [23]. Collectively, these studies enrolled 8,806 patients, of whom 2915 underwent no axillary surgery and 5891 received axillary surgical intervention. Five studies compared the omission of axillary surgery with axillary lymph node dissection (ALND), and two studies compared it with sentinel lymph node biopsy (SLNB) (Table 1). Follow-up durations ranged from 5 to 25 years. Most trials included clinically node-negative women undergoing breast-conserving surgery, although some allowed mastectomy.

**Table 1:** Characteristics of included studies

	Patient enrollment period	N	Axillary surgery	Age	Tumor size	Breast surgery	Axillary evaluation	Adjuvant therapy
INSEMA (Reimer et al., 2024)	2015–2019	4858	SLNB	> 18 years	T1/T2	BCS	US	Whole-breast RT (no axillary RT); CT in ~ 10–13%; presumed HT (95% HR +); no data on HER2 therapies
SOUND (Gentillini et al., 2023)	2012–2017	1405	SLNB	Any age	T1	BCS	US	RT in > 97% (mostly whole-breast); CT in ~ 18–20%; HT in ~ 98% of HR + ; trastuzumab in ~ 94–98% of HER2 +
INT 09/98 (Agresti et al., 2024)	1998–2003	517	ALND	< 65 years	T1/T2	BCS	Clinical exam	RT to breast/chest wall ± nodes; CT in 100%; HT in 56% (tamoxifen); no HER2 data
Martelli et al., 2014	1996–2000	219	ALND	65–80 years	T1	BCS	Clinical exam	RT in 63% (post-BCS); no CT; HT with tamoxifen (97%); no HER2 data
AXIL95 (Avril et al., 2012)	1995–2005	607	ALND	> 45 years	T1a/T1b	BCS	Clinical exam	RT to breast/chest wall ± supraclavicular; CT in ~ 74%; HT in 75% (tamoxifen); no HER2 data
IBCSG 10–93 (Rudenstam et al., 2005)	1993–2002	473	ALND	> 60 years	T1	BCS	Clinical exam	RT in ~ 33% (post-BCS); no CT; tamoxifen 5y (96%; 80% HR +); no HER2 data
NSABP B04 (Fisher et al., 2002)	1971–1974	727	ALND	Any age	T1/T2/T3	Mastectomy	Clinical exam	No RT; no CT; no HT; surgery-only trial

*ALND* axillary lymph node dissection, *BCS* breast-conserving surgery, *CT* chemotherapy, *HT* hormone therapy, *RT* radiotherapy *SLNB* sentinel lymph node biopsy

### Disease-free survival (DFS)

**Fig. 2:**
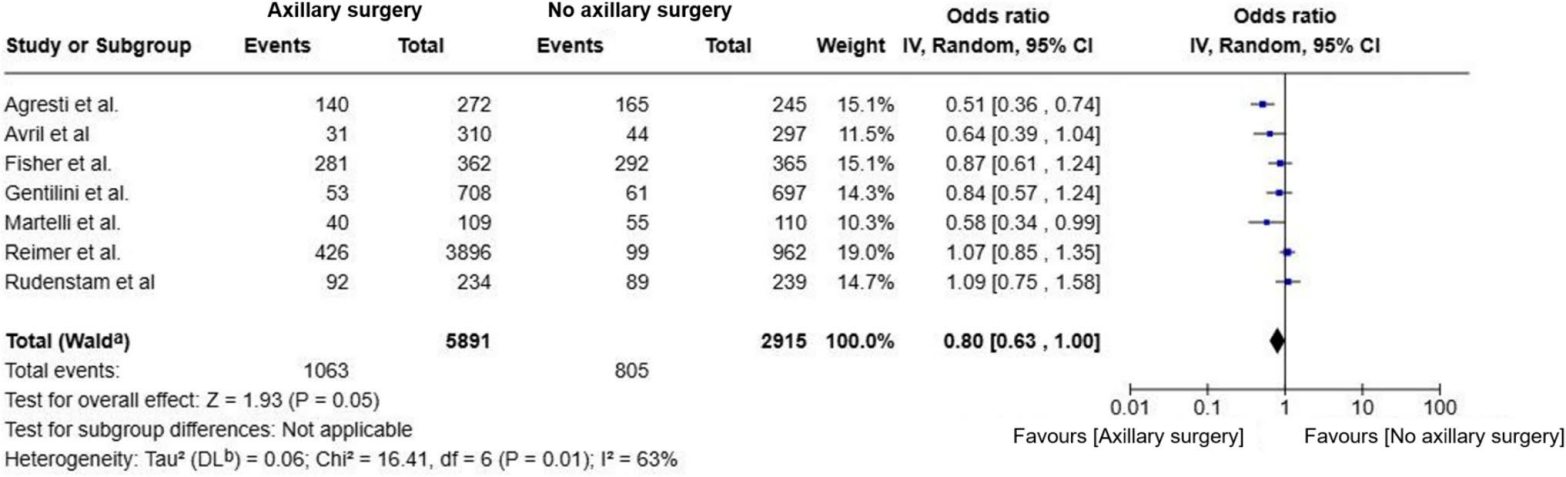
Forest plot of disease-free survival in patients comparing omission of axillary surgery to axillary surgery (ALND or SLNB)

Meta-analysis showed no statistically significant difference in DFS between the no-surgery and axillary surgery groups (OR = 0.80; 95% CI, 0.63–1.00; p = 0.05; I^2^ = 63%) (Fig. 2). While the effect size slightly favored axillary surgery, the confidence interval included the threshold of statistical significance, warranting cautious interpretation. Moderate to high heterogeneity was observed, likely due to variations in DFS definitions and follow-up durations.

**Fig. 3:**
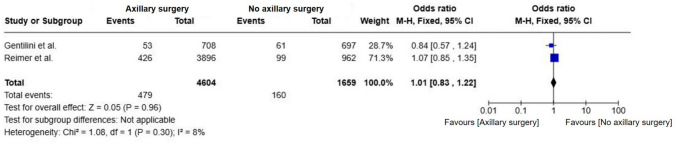
Forest plot of disease-free survival in patients from the reimer and gentilini trials comparing omission of axillary surgery to sentinel lymph node biopsy

As methodological and statistical heterogeneity was noted between most studies and the Reimer et al., 2024 [17] and Gentilini et al., 2023 [18] trials, a subgroup analysis was prepared with these two studies and, no significant difference in DFS was observed between omission of axillary surgery and SLNB (OR = 1.01; 95% CI, 0.83–1.22; p = 0.96; I^2^ = 8%) (Fig. 3). The low heterogeneity highlights the methodological consistency between these studies.

### Overall survival (OS)

**Fig. 4:**
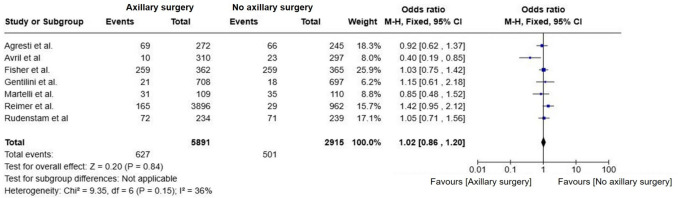
Forest plot of overall survival in patients comparing omission of axillary surgery to axillary surgery (ALND or SLNB)

**Fig. 5:**
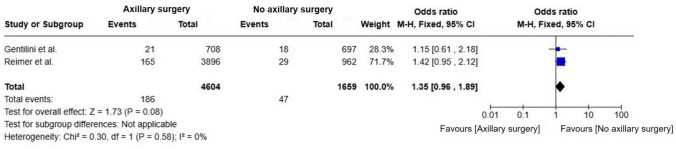
Forest plot of overall survival in patients from the reimer and gentilini trials comparing omission of axillary surgery to sentinel lymph node biopsy

There was no significant difference in OS between groups (OR = 1.02; 95% CI, 0.86–1.20; p = 0.84; I^2^ = 36%) (Fig. 4). When restricting the analysis to the Reimer et al., 2024 [17] and Gentilini et al., 2023 [18] trials, no statistically significant difference in OS was observed (OR = 1.35; 95% CI, 0.96–1.89; p = 0.08; I^2^ = 0%) (Fig. 5). The absence of heterogeneity reinforces the consistency of findings across both studies. Although the point estimate slightly favored axillary surgery, the confidence interval crossed unity.

### Axillary recurrence (AR)

**Fig. 6:**
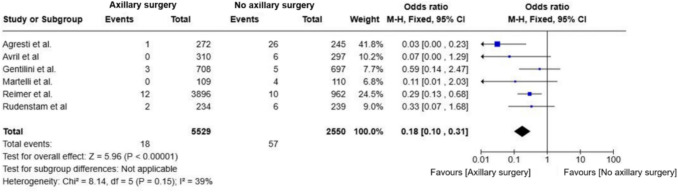
Forest plot of axillary recurrence in patients comparing omission of axillary surgery to axillary surgery (ALND or SLNB)

Patients in the omission group had a significantly higher risk of axillary recurrence (OR = 0.18; 95% CI, 0.10–0.31; p < 0.01; I^2^ = 39%) (Fig. 6). Despite this, absolute recurrence rates were low (< 2%). The consistent direction of effect reinforces the potential role of axillary surgery in reducing locoregional recurrence. Notably, the highest recurrence rates were observed in older trials lacking standardized radiotherapy protocols and routine preoperative ultrasound.

**Fig. 7:**
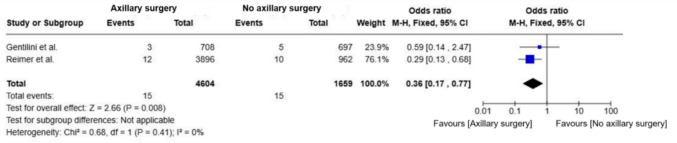
Forest plot of axillary recurrence in patients from the reimer and gentilini trials comparing omission of axillary surgery to sentinel lymph node biopsy

In the subgroup analysis of the Reimer et al., 2024 [17] and Gentilini et al., 2023 [18] trials, the pooled odds ratio for axillary recurrence favored SLNB (OR = 0.36; 95% CI, 0.17–0.77; p = 0.008), with no observed heterogeneity (I^2^ = 0%) (Fig. 7). Absolute recurrence rates remained low in both groups.

### Risk of bias

**Fig. 8:**
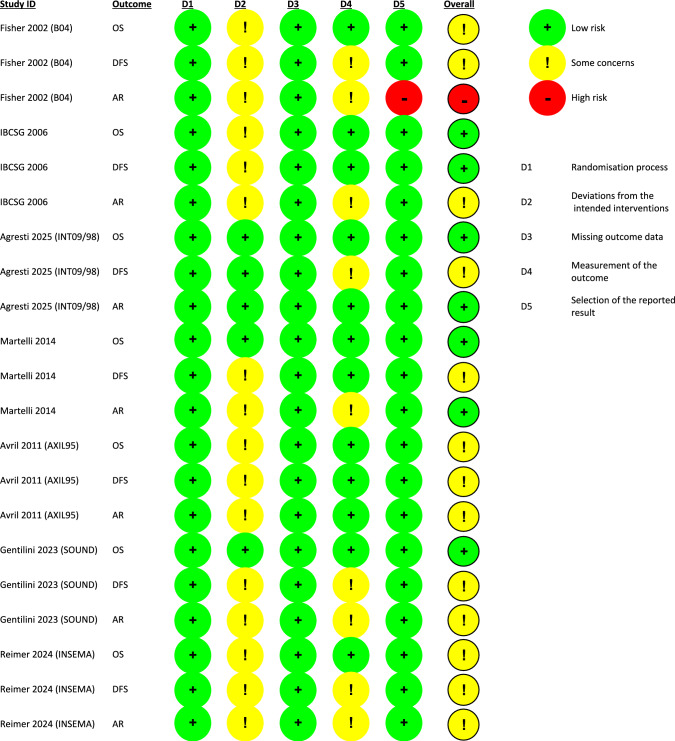
Assessment of the risk of bias for each of the three outcomes of the randomized clinical trials, conducted using the RoB 2 tool

Two independent reviewers assessed the risk of bias across five domains using the Cochrane RoB 2 tool. Discrepancies were resolved by a third reviewer. Each domain was evaluated for all reported outcomes (OS, DFS, and AR) and classified as low risk, some concerns, or high risk of bias (Fig. 8).

### Randomization process

All seven studies were described as randomized and reported adequate randomization procedures. Baseline characteristics were well-balanced between groups. Consequently, all studies were judged to have a low risk of selection bias.

### Deviations from intended interventions

Blinding of participants and providers was not feasible due to the surgical nature of the interventions. The absence of blinded outcome assessment may have influenced DFS and AR outcomes. Some concerns were noted in studies that did not clearly describe the methods used for axillary follow-up. OS was considered less susceptible to bias from lack of blinding and was generally assessed as low risk. However, some concerns were raised for OS in Reimer et al., 2024 and Avril et al., 2012 due to a higher use of adjuvant chemotherapy in the surgery group. In Fisher et al., 2002, one-third of patients in the no-surgery group had lymph nodes identified in mastectomy specimens—an unintended deviation that led to concerns of bias across all outcomes.

### Missing outcome data

All studies reported outcome data for nearly all randomized participants and were deemed low risk in this domain. However, Gentilini et al., 2023; Avril et al., 2012; and Rudenstam et al., 2005 did not reach their planned sample sizes.

### Measurement of the outcome

Due to the unblinded nature of the trials, there may have been more intensive follow-up or additional interventions in the no-surgery group, potentially influencing DFS and AR. This led to some concerns of bias for these outcomes in several studies.

### Selection of the reported result

Primary outcomes were adequately reported in all studies except Fisher et al., 2002, which did not report AR rates for the surgery group. This was judged to represent a high risk of bias for that specific outcome.

## Discussion

This systematic review of seven randomized clinical trials demonstrated no significant differences in disease-free survival (DFS) or overall survival (OS) between women with breast cancer who underwent axillary surgery and those who did not. However, several important considerations must be acknowledged when interpreting these findings.

In the two most recent trials [17, 18], axillary intervention consisted of sentinel lymph node biopsy (SLNB), and axillary ultrasound (US) was employed for patient selection. In contrast, the earlier trials used axillary lymph node dissection (ALND) as the surgical intervention, with patient selection based on clinical examination alone [19–23]. This difference increased the statistical heterogeneity within the forest plot analyzing the outcomes and also presented methodological heterogeneity, rationale for the use of subgroup analysis. The false-negative rate of axillary US in the recent studies was approximately 13–15% in the SLNB arms—an important limitation that underscores the need for standardized axillary US protocols when considering omission of axillary surgery.

The earliest trials [22, 23] included patients with cT1–cT3 tumors, treated with either breast-conserving surgery followed by radiotherapy or mastectomy. All subsequent studies focused primarily on breast-conserving surgery with whole-breast irradiation and no specific axillary treatment. However, as reported in the Reimer trial [18], more than 50% of patients received at least 80% of the prescribed radiotherapy dose to axillary level I, which may have contributed to local control. Additionally, patient selection in most studies favored postmenopausal women with stage I tumors and, in nearly all cases, luminal-like immunohistochemical profiles.

The rate of positive lymph nodes among patients undergoing axillary intervention ranged from 13 [17] to 28% [19]. Notably, axillary recurrence rates in the no-surgery arms showed a decreasing trend over time. Reported axillary recurrence rates were 18.6% in Fisher et al. and 10.6% in Agresti et al. [19], with follow-up periods of 25 and 21 years, respectively. In Martelli [20], Rudenstam [22], and Avril [21], recurrence rates were 3.6, 2.5, and 2.0%, with follow-up of 15, 6, and 5 years. In the most recent trials—Reimer et al., 2024, and Gentilini et al., 2023—where the comparator was SLNB, recurrence rates were 1.0 and 0.7%, respectively, with mean follow-up durations of 6 and 5 years, suggesting improvements in patient selection with ultrasound inclusion and the increasing impact of modern systemic therapies. In Fisher et al. [23], treatment consisted solely of surgery and radiotherapy, while more recent trials included systemic therapy tailored to tumor biology.

Our meta-analysis found that omission of routine axillary dissection in clinically node-negative early breast cancer results in a small but statistically significant increase in axillary recurrences while not producing measurable differences in disease-free or overall survival. A plausible biological explanation is that axillary lymph nodes primarily serve as a sentinel indicator of a tumor’s metastatic potential rather than the principal source of future systemic spread. By the time microscopic tumor cells reach regional nodes, dissemination to distant sites may already have occurred in the subset of biologically aggressive tumors; conversely, many nodal metastases may be biologically indolent and insufficient to establish distant metastases. In addition, distance metastasis may occur without the presence of axillary metastasis. Therefore, removing additional nodal tissue does not necessarily reduce the risk of distant relapse or death.

Although the short follow-up in the most recent studies raises some uncertainty regarding the durability of these low recurrence rates, they are expected to remain low over time. This expectation is based on the typically indolent course of axillary recurrence in luminal A-like tumors, which often takes many years to manifest. Nonetheless, even low recurrence rates warrant attention. Agresti et al. [19], for example, showed that patients with biologically unfavorable tumors experienced earlier recurrences and higher rates of distant metastasis, despite the lack of significant differences in DFS and OS.

Importantly, at least five of the included studies had a limitation regarding the underrepresentation of tumor subtypes other than luminal A-like, as well as younger patients and those with cN0 status following neoadjuvant systemic therapy. Ongoing randomized trials—such as VENUS [14], BOOG 2013–08 [15] and NAUTILUS [16]—may help address some of these gaps. This is particularly relevant because axillary status remains a key factor in therapeutic decision-making for many patients, especially those with distinct clinical and pathological features not adequately represented in current trials.

The limitations of our study are primarily related to the heterogeneity among the included trials. These studies varied substantially in key parameters, including the year of publication (ranging from 1970 to 2024) and duration of follow-up (from 5 to 25 years). Such variability complicates the interpretation of pooled results and limits the generalizability of conclusions. To mitigate this, we conducted a focused analysis of the two most methodologically homogeneous studies, which shared similar inclusion criteria and follow-up periods. The results of this review might suggest that omission of axillary surgery is more focused in clinically and ultrasonographically negative axillary lymph nodes, and less aggressive breast tumors. This can be reinforced by the low risk of bias for overall survival of the SOUND study and unclear risk of bias by the INSEMA study. Additionally, several breast cancers subtypes and clinical scenarios remain underrepresented in the current literature. This limits the extrapolation of findings to broader populations, as it remains uncertain whether the observed outcomes are applicable to patients with more aggressive subtypes or those undergoing neoadjuvant therapy or mastectomy.

## Conclusion

In recent randomized trials, axillary recurrence was slightly more frequent in patients who did not undergo axillary surgery; however, it remained a rare event overall. Importantly, the omission of axillary surgery did not compromise overall survival or disease-free survival. These results, however, should be interpreted with caution in underrepresented subgroups such as younger patients, T2 or T3 tumors, individuals with HER2-positive tumors, and those with triple-negative breast cancer.

## Data Availability

No datasets were generated or analysed during the current study.
